# Statistical analysis plan for the 24-week randomised trial of hypoglycaemia prevention, awareness of symptoms, and treatment: HypoPAST

**DOI:** 10.1016/j.conctc.2025.101513

**Published:** 2025-07-02

**Authors:** Sharmala Thuraisingam, Jennifer A. Halliday, Uffe Søholm, Elizabeth Holmes-Truscott, Christel Hendrieckx, Timothy C. Skinner, Vincent L. Versace, Jane Speight, HypoPAST study group

**Affiliations:** a Deakin Rural Health, School of Medicine, Faculty of Health, Deakin University, Warrnambool, Victoria, 3280, Australia; b School of Psychology, Deakin University, 1 Gheringhap St, Geelong, Victoria, 3220, Australia; c The Australian Centre for Behavioural Research in Diabetes, Diabetes Victoria, 15-31 Pelham Street, Carlton, Victoria, 3053, Australia; d Institute for Health Transformation, Deakin University, 1 Gheringhap St, Geelong, Victoria, 3220, Australia; e Institute of Psychology, Copenhagen University, Copenhagen, Denmark; f Geohealth Laboratory, Department of Population Health, Dasman Diabetes Institute, Kuwait City, Kuwait

**Keywords:** Statistical analysis plan, Randomised controlled trial, Type 1 diabetes, Hypoglycaemia, Online psycho-educational training

## Abstract

**Background:**

The HypoPAST (Hypoglycaemia Prevention, Awareness of Symptoms, and Treatment) randomised controlled trial aims to examine the effectiveness of an online psycho-educational intervention for reducing fear of hypoglycaemia among adults with type 1 diabetes. This statistical analysis plan provides the framework to assess the primary, secondary, and safety outcomes of the trial. The plan was written prior to database lock and in accordance with the SPIRIT guidelines.

**Methods:**

HypoPAST is a 24-week, two-arm, parallel-group, hybrid type 1 randomised controlled trial. The primary outcome is the difference in mean Hypoglycaemia Fear Survey II Worry subscale scores at 24 weeks between intervention and control arms. Secondary outcomes include between-arm differences in psychological, clinical and behavioural measures at mid- and end-trial. Primary and secondary outcomes will be analysed using mixed-effects models under the intention-to-treat principle. A sensitivity analysis will examine assumptions regarding missing data, and a per-protocol analysis will estimate the intervention effect among participants who engage with HypoPAST. Table shells for all prespecified analyses are provided to support transparent reporting.

**Conclusion:**

Consistent with best practice, all analyses described were prespecified prior to completion of trial data collection. The analysis methods were developed by statisticians, with input from trial investigators. This analysis plan provides a rigorous framework for the analysis of the HypoPAST trial data, ensuring the results will be robust and reproducible.

**Trial registration:**

The trial is registered on the Australian and New Zealand Clinical Trials Registry: ACTRN12623000894695 (August 21, 2023).

## 1 Background

Type 1 diabetes is a chronic condition, due to the immune system destroying the beta cells in the pancreas that produce insulin [1]. This results in hyperglycaemia (high blood glucose levels), which requires ongoing, intensive management with exogenous insulin. Despite recent advancements in insulin therapy and delivery, injecting exogenous insulin does not mimic endogenous insulin supply [1]. Hypoglycaemia (low blood glucose) is a common side-effect of insulin treatment, due to excess insulin relative to blood glucose. It can be life-threatening if undetected and untreated [2,3].

Approximately 20 % of people with type 1 diabetes report experiencing severe hypoglycaemia (defined as requiring assistance from someone else for recovery) in the past 6 months [4]. Symptoms of hypoglycaemia are typically unpleasant, including sweating, heart palpitations, confusion, shaking, difficulty speaking and coordinating movements [2]. These can be unpredictable, sudden and embarrassing, resulting in hypoglycaemia-related anxiety, known as fear of hypoglycaemia [5].

Advanced automated insulin delivery and glucose monitoring systems may help reduce some, but not all, hypoglycaemia – and do not necessarily alleviate fear of hypoglycaemia [6,7]. Group-based, face-to-face psycho-educational and behavioural training programs focus on improving awareness of, and timely, appropriate responses to, decreasing glucose levels, and have demonstrated benefits in reducing fear of hypoglycaemia [5,8,9]. However, adoption of these programs as part of routine clinical care in Australia is low due to the resources required for their delivery.

HypoPAST (Hypoglycaemia Prevention, Awareness of Symptoms, and Treatment) is a self-directed, fully-online, psycho-educational program, designed to reduce fear of hypogylcaemia. The aim of the HypoPAST randomised controlled trial (RCT) is to assess the effectiveness of the HypoPAST program in reducing fear of hypoglycaemia at 24 weeks among adults with type 1 diabetes, compared with usual care [10]. This statistical analysis plan details the methods that will be used to assess the primary, secondary (clinical, psychological, and behavioural) and safety outcomes of the HypoPAST RCT.

## 2 Methods

This study is a 24-week, two-arm, parallel-group, hybrid type 1 randomised controlled trial assessing the effect of HypoPAST, a fully-online psycho-educational program aiming to reduce fear of hypoglycaemia in adults with type 1 diabetes. The study has four objectives:

1. To assess the effect of HypoPAST on fear of hypoglycaemia among adults with type 1 diabetes;
2. To assess the effect of HypoPAST on secondary psychological, clinical and behavioural outcomes;
3. To assess the cost-effectiveness of HypoPAST; and
4. To explore the reach, acceptability, usability and sustainability of HypoPAST to adults with type 1 diabetes, and understand the extent to which learnings from the programme are implementable in the ‘real-world’, using a mixed-methods process evaluation.

The RCT protocol is published in full elsewhere [10]. Ethics approval was granted by Deakin University Human Research Ethics Committee (ref: 2023–132).

This manuscript reports the statistical analysis plan for Objectives 1 and 2, with specific focus on analysis of clinical, psychological, and behavioural outcome data. Analysis of other trial data (for Objectives 3–4) are beyond scope of this manuscript; refer to the published protocol [10] for additional information about these.

### 2.1 Inclusion criteria

Adults (aged 18+ years) will be eligible to participate if they live with type 1 diabetes, in Australia, and report hypoglycaemia-related worries. A single item on the PAID scale [11], worry about low blood glucose, will be used to determine hypoglycaemia-related worry at recruitment. Participants who report this as a “moderate”, “somewhat serious” or “serious” problem will be eligible.

### 2.2 Primary outcome

Difference in mean Hypoglycaemia Fear Survey II (HFS-II) Worry score at trial end (week 24) between the intervention and control arms.

### 2.3 Secondary outcomes

The secondary outcomes and measures relevant to this statistical analysis plan are summarised in Table 1. For (normally distributed) continuous outcomes, the between-arm difference in mean outcome will be estimated at mid-trial (week 12) and trial-end (week 24). For count data, the ratio of incident rates between trial arms will be estimated at mid-trial and trial end. Kruskal-Wallis tests will be used to assess possible differences between the trial arms for ordinal variables (at 12 and 24 weeks).

**Table 1:** Secondary outcomes.

Outcome measure	Type	Effect estimate
***Glucose levels, targets, and monitoring***		
Most recent HbA1c (% or mmol/mol)	Continuous	Difference in mean
Target glucose lower and upper limits (mmol/L)	Continuous	Difference in mean
Time below and above target range (%; CGM/isCGM users only)	Continuous	Difference in mean
Comfortable blood glucose lower and upper limits (mmol/L)	Continuous	Difference in mean
Daily number of glucose checks	Ordinal	η^2^ Kruskal-Wallis test

***Insulin use***		
Daily number of insulin doses/boluses	Ordinal	η^2^ Kruskal-Wallis test
Daily number of insulin units	Count	Incident rate ratio

***Awareness of hypoglycaemia symptoms***		
Gold score [12]	Continuous	Difference in mean
Technological awareness study-specific question (1 item)	Continuous	Difference in mean
HypoA-Q 5-item Impaired Awareness subscale score [13]	Continuous	Difference in mean

***Hypoglycaemia symptom burden and response***		
Study-specific questions (2 items)	Ordinal	η^2^ Kruskal-Wallis test

*H****ypoglycaemia frequency & severity***		
HypoA-Q items 1–4 [13]	Ordinal	η^2^ Kruskal-Wallis test
HypoA-Q items 15 and 16 [13]	Count	Incident rate ratio

***Behaviours associated with fear of hypoglycaemia***		
HFS-II SF Avoidance subscale scores [14]	Continuous	Difference in mean
HFS-II SF Maintain High subscale scores [14]	Continuous	Difference in mean

***Confidence in managing hypoglycaemia***		
Hypoglycaemia Confidence Scale score [15]	Continuous	Difference in mean

***Hypoglycaemia-specific quality of life***		
HIP-12 composite score [16]	Continuous	Difference in mean

***Attitudes to awareness of hypoglycaemia***		
A2A Asymptomatic Hypoglycaemia Normalised scale score [17]	Continuous	Difference in mean
A2A Hyperglycaemia Avoidance Prioritised scale score [17]	Continuous	Difference in mean
A2A Hypoglycaemia Concern Minimised scale score [17]	Continuous	Difference in mean
Study-specific questions (4 items)	Ordinal	η^2^ Kruskal-Wallis test

***Perceptions and experiences of hypoglycaemia***		
HypoC-Q Low concern about hypoglycaemia scale score [18]	Continuous	Difference in mean
HypoC-Q Hypoglycaemia burnout scale score [18]	Continuous	Difference in mean
HypoC-Q Missing Opportunities to Treat Hypoglycaemia scale score [18]	Continuous	Difference in mean
HypoC-Q Delaying treatment of hypoglycaemia scale score [18]	Continuous	Difference in mean
HypoC-Q individual questions scored separately (12 items) [18]	Ordinal	η^2^ Kruskal-Wallis test

***Hypoglycaemia-specific post-traumatic stress***		
PC-PTSD-5a total score [19]	Continuous	Difference in mean

***Diabetes distress***		
PAID-11 scale score [11]	Continuous	Difference in mean

***Anxiety and depressive symptoms***		
PHQ-4 2-item Anxiety subscale [20]	Continuous	Difference in mean
PHQ-4 2-item Depressive symptoms subscale [20]	Continuous	Difference in mean

Abbreviations: HbA1c = glycated haemoglobin, isCGM = intermittently scanned continuous glucose monitoring, CGM = continuous glucose monitoring, HypoA-Q = Hypoglycaemia Awareness Questionnaire, HFS-II SF=Hypoglycaemia Fear Survey II Short Form, HIP-12 = Hypoglycaemia Impact Profile, 12 items, A2A = Attitudes to Awareness of Hypoglycaemia, HypoC-Q = Hypoglycaemia Cues Questionnaire, PC-PTSD-5a = Primary Care Post-Traumatic Stress Disorder 5 items, PAID-11 = Problem Areas in Diabetes, 11 items, PHQ-4 = Patient Health Questionnaire, 4 items.

Note: All outcomes assessed at 12 and 24 weeks.

### 2.4 Safety outcomes

Serious adverse events (SAEs) are any severe hypoglycaemic or mental-related health event occurring during the study for which the participant required medical assistance (emergency services were called, the participant was taken to an emergency department or admitted to hospital).

### 2.5 Intervention

HypoPAST (Hypoglycaemia Prevention, Awareness of Symptoms, and Treatment) is a fully online psycho-educational program. Participants begin by completing a brief ‘Getting Started’ module, which demonstrates how to make the most of the program and enables participants, via self-assessment, to prioritise the modules most relevant to their needs. Next, they gain access to seven modules (30–60 min each) about topics known to contribute to fear or worries about hypoglycaemia (e.g. severe hypoglycaemia, reactions from other people, sleep), ordered according to their self-assessment. The modules include self-guided psychoeducational activities, videos, and printable resources, specifically designed to support adults with type 1 diabetes to strengthen their skills in preventing and managing fear of hypoglycaemia. These are informed by 30 years of research into group-based, hypoglycaemia-specific psycho-educational training programs [21]; behavioural, psychological, educational and clinical insights from a multidisciplinary team; and lived experience insights from a Steering Group of adults with type 1 diabetes [10]. In total, the program is estimated to take 4–8 h, depending on how many modules the participant accesses, and how long they spend on activities and practising skills between modules. HypoPAST offers potential benefits over and above existing hypoglycaemia-specific psycho-educational programs, which are typically group-based, face-to-face, multi-week and require facilitation by a health professional. These include a self-directed, self-paced fully online training program, enabling equitable access and minimal health system costs [10].

Intervention participants will have access to all HypoPAST modules throughout the 24-week trial period but will be encouraged to work through the modules within 4–8 weeks of being randomised to enable sufficient time for digesting information, completing ‘experiments’, self-reflection on, and change in, hypoglycaemia experiences throughout the remaining weeks.

Control arm participants will be advised to engage in their usual diabetes self-management. This is assumed to include strategies to minimise risk of problematic hypoglycaemia and contact with their diabetes specialist or healthcare team, and may also include contact with the National Diabetes Services Scheme (NDSS) Helpline. At completion of the trial, control arm participants will be offered access to HypoPAST for 24 weeks.

### 2.6 Randomisation and blinding

Participants meeting the study inclusion criteria and who have completed all baseline data collection requirements will be randomised to the intervention or control arms in a 1:1 ratio, using randomly permuted block sizes of 4 and 6. Randomisation of participants will be coded in Qualtrics [22] using randomly generated allocation sequence tables and piped to Platform O, a Deakin-owned and hosted e-Research tool. The Qualtrics platform will automatically generate the allocation sequence using its internal randomisation algorithm. The process will be fully automated, with no involvement from the trial statistician or study personnel, ensuring allocation concealment. Prior to randomisation, participants will be stratified by gender and glucose monitoring method, i.e. self-monitoring of blood glucose alone or continuous glucose monitoring (CGM) – noting that CGM includes intermittently scanned methods (isCGM). Participants that do not identify as either male or female will be randomly allocated to either the male or female strata to ensure an even representation of this subgroup in both these strata.

The HypoPAST researchers will be blinded to participant trial arm allocations and identifiers. Only a research assistant (responsible for maintaining a participant codebook and email communication with participants) and the website architect of Platform O will be aware of group allocations. To maintain blinding, neither of these staff will have access to the Qualtrics surveys during data collection or be involved in data analysis.

### 2.7 Sample size

To date, there is no published consensus on a minimal clinically important difference in fear of hypoglycaemia. Hence, prior research was used to estimate a clinically important effect size [18]. To detect a between-arm difference of nine points (standardised effect size of 0.5) in the primary outcome at end-trial, a total of N = 150 participants (n = 75 per arm) is required, assuming a standard deviation of 17 [23], 90 % power, 5 % significance level, 1:1 trial arm allocation for a two-sided test. As the exact attrition rate was unknown at the time of planning, we explored a range of inflation factors based on plausible scenarios, recruitment feasibility, and evidence from similar studies [24]. Based on this, a 30 % inflation factor was applied, resulting in a final required sample size of N = 196 participants (n = 98 per trial arm) [25]. This sample size is a conservative estimate based on a two-sided test, high level of power and allowance for attrition. All sample size calculations were conducted using STATA MP version 17.0 (StataCorp) [26].

### 2.8 Data collection and preparation

All clinical (including HbA1c, target glucose range), psychological, and behavioural data, will be collected via self-report online surveys hosted on Qualtrics [22]. All participants will complete demographic questions at baseline (pre-randomisation), and primary and secondary outcome measures at baseline, mid-trial (week 12) and end-trial (week 24). Survey questions will be tailored (using survey ‘logic’ condition settings) for relevance to the participant's diabetes management (for example, only participants using isCGM/CGM will be asked to report percentage of time above/below range).

At each time point, automated emails will provide participants with a link to the survey for completion. Automated reminders to complete the surveys will be emailed one and two weeks after each initial survey invitation is sent. Participants can choose to skip survey questions they do not want to answer.

At the end of the trial, all participant identifiers will be removed, and trial arms renamed randomly by the research assistant to either arm A or B to ensure the study statistician is blinded to arm allocations during analyses. True arm allocations will be revealed at the completion of reporting of all results. Coding of the primary and secondary outcomes will be consistent with Appendix A. Supplementary File 1, Table 1.

### 2.9 Statistical analysis

#### 2.9.1 Trial profile

**Fig. 1:**
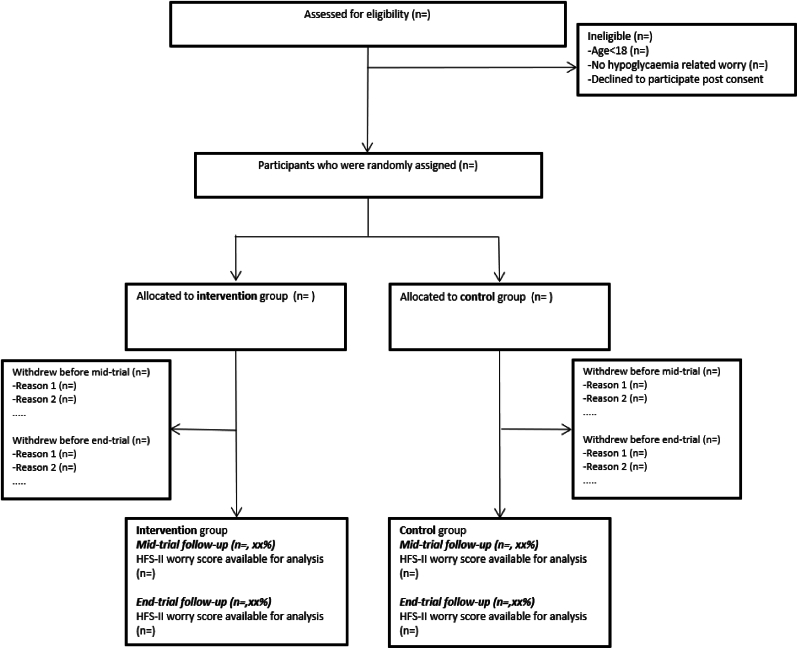
Trial flowchart.

A study flow diagram (Fig. 1) consistent with CONSORT guidelines [27] will be used to summarise the number of participants assessed for eligibility, randomised and analysed.

#### 2.9.2 Descriptive statistics

All analyses will be conducted using STATA MP version 18.0 (StataCorp) [28]. Participant socio-demographic and clinical characteristics will be summarised at baseline and presented as shown in Table 2. The mean and standard deviation will be used to summarise approximately normally distributed continuous data, and frequencies and percentages for categorical data. Skewed continuous variables will be summarised using the median and interquartile range. Missing data will be summarised using counts and percentages.

**Table 2:** Participant baseline characteristics.

		Total (N = )	Intervention (N = )	Control (N = )
***Socio-demographics characteristics***				
Age *(years)*	Mean (SD)			
Gender	n (%)			
Man or male				
Woman or female				
Non-binary				
Other				
Prefer not to answer				
Missing				
Aboriginal or Torres Strait Islander	n (%)			
Aboriginal				
Torres Strait Islander				
Both Aboriginal and Torres Strait Islander				
No				
Prefer not to say				
Missing				
Language spoken at home	n (%)			
English				
English and other				
Other only				
Missing				
Country of birth	n (%)			
Australia				
Other				
Missing				
State	n (%)			
Victoria				
New South Wales				
South Australia				
Australian Capital Territory				
Queensland				
Tasmania				
Western Australia				
Northern Territory				
Missing				
Highest qualification completed	n (%)			
High school or lower				
Certificate 1–4 or trade certificate				
Diploma, Advanced diploma or Associate degree				
Bachelor degree, Honours, Graduate certificate or Graduate diploma				
Masters or doctoral degree				
Missing				
Current employment	n (%)			
Paid full-time				
Paid part-time				
No paid work but seeking paid work				
No paid work and not seeking paid work				
Missing				
Do you live with another person who can assist with a hypo?	n (%)			
Yes				
No				
Missing				
Relationship status	n (%)			
Married or De-Facto (living together)				
In a relationship (but not living together)				
Single (including widowed, separated, divorced, dating or not dating)				
Missing				
Previous diabetes education	n (%)			
Yes				
None				
Missing				
Time of main sleep				
Night				
Day				
Missing				
***Clinical characteristics***				
Type 1 diabetes duration (years)	Mean (SD)			
Missing				
Diabetes-related complications (ever)	n (%)			
None				
One or more				
Missing				
History of severe hypoglycaemia	n (%)			
Main insulin administration method	n (%)			
Multiple daily insulin injections				
Insulin pump				
Automated insulin delivery (commercial brand)				
Automated insulin delivery (open-source)				
Missing				
Main glucose monitoring modality	n (%)			
Finger pricks (self-monitoring of blood glucose; SMBG)				
Continuous Glucose Monitoring (CGM)				
Intermittently scanned CGM (isCGM)				
Missing				

Abbreviations: SD = standard deviation.

Diabetes-related complications include: Heart attack or heart disease, stroke, retinopathy, kidney disease, renal failure, nephropathy, peripheral vascular disease, peripheral neuropathy, autonomic neuropathy, sexual dysfunction, erectile dysfunction, impotence, gingivitis or periodontitis, other.

#### 2.9.3 Main analysis

An intention to treat (ITT) approach will be used, whereby all randomised participants will be included in the analysis and their data analysed according to trial arm assignment. A linear mixed model using restricted maximum likelihood estimation will be used to estimate trial arm differences in the primary outcome (HFS-II Worry score) at 24 weeks. HFS-II Worry score at 12 and 24 weeks will be included in the model as the dependent variables. Fixed effects in the model will include time (12 and 24 weeks), HFS-II Worry score at baseline, and an interaction between time and trial arm. Repeated participant outcome measures will be accounted for as random effects in the model using an unstructured variance-covariance structure given correlations in the outcome within individuals are expected to be unique. The model will be adjusted by two stratification factors: gender and glucose monitoring method. Should the distribution of the HFS-II Worry score be skewed, transformations will be considered. The estimated mean HFS-II Worry score at baseline, 12 and 24 weeks will be plotted for each trial arm with 95 % confidence intervals. The mean difference in HFS-II Worry score between arms at 24 weeks (and 12 weeks) will be reported along with 95 % confidence intervals and p-values as shown in Table 3.

**Table 3:** Estimated trial arm differences from main, adjusted, sensitivity and per protocol analyses.

	Intervention (N = )		Control (N = )		Intervention vs control between-arm differences (95 % CI)	P-value
	n	Estimated mean (95 % CI)	n	Estimated mean (95 % CI)		
Fear of hypoglycaemia – HFS-II Worry scale score						
*Main analysis*						
Baseline						
Mid-trial: 12 weeks						
End-trial: 24 weeks					a	
*Adjusted analysis*						
Baseline						
Mid-trial						
End-trial						
*Sensitivity analyses – multiple imputation*						
Baseline						
Mid-trial						
End-trial						
*Per protocol analysis*						
Baseline						
Mid-trial						
End-trial						

Per protocol analysis: engaged intervention participants defined as having used at least two of the seven training modules.

Note: Main analysis adjusted by stratification factors (gender and glucose monitoring method). Adjusted analysis includes further adjustment by potential prognostic factors (age, diabetes duration, HbA1c, severe hypoglycaemia in the last 6 months, Gold score and insulin administration modality).

Abbreviations: CI = confidence interval.

a Primary outcome.

#### 2.9.4 Adjusted analysis

The primary outcome will be adjusted by potential prognostic factors: age, diabetes duration, HbA1c, severe hypoglycaemia in the past 6 months, Gold score and insulin administration modality. Adjusted regression results will be displayed alongside estimates from the main analysis (Table 3).

#### 2.9.5 Sensitivity analyses

A sensitivity analysis using multiple imputation will be conducted on the primary outcome should there be between 10 % and 40 % missing data in the primary outcome and auxiliary variables available in the data set to explain the missingness. This threshold is based on a rule of thumb and reflects current guidance that multiple imputation generally performs well with moderate missingness but may be less reliable when missingness exceeds approximately 40 % [29,30]. Differences in mean HFS-II Worry score, 95 % confidence intervals and p-values will be presented as shown in Table 3. The intervention effect estimate will be included in a forest plot.

A second sensitivity analysis will be conducted on the primary outcome to test the robustness of the missing at random (MAR) missing data assumption of linear mixed models. Pattern-mixture models [31] will be used to determine whether study conclusions would change if the missing data were missing not at random (MNAR).

Under the MAR assumption, the difference in the mean of the missing data and mean of the observed data, δ, is assumed to be zero. In the pattern-mixture models, a range of plausible negative and positive values for δ will be considered. Positive values of δ indicate that participants with missing data have on average higher (worse) HFS-II Worry scores than observed participants [31]. The opposite is true for negative values of δ. To ensure the range of δ values reflects clinically and contextually meaningful scenarios, experts in the field will be consulted to help define plausible values for δ. Where available, external data or prior studies will also inform these values. The chosen δ values will span from zero (consistent with MAR) to positive and negative values representing plausible departures, anchored to the outcome scale. Regression results for a range of plausible values of δ will be used to determine whether study conclusions would change if the missing data were not assumed to be MAR. Results will be displayed in a graph with estimated differences in mean HFS-II Worry score and 95 % confidence intervals plotted against plausible values of δ and included in the appendices of the main trial outcomes paper.

#### 2.9.6 Per protocol

A per protocol analysis for the primary outcome will be conducted to estimate the treatment effect in those who engage with the intervention. Engagement will be defined as having used at least two of the seven training modules. The effect estimates, 95 % confidence intervals and p-values obtained from the per protocol analysis will be included in Table 3. The effect estimate will be included in a forest plot alongside estimates obtained from the main and adjusted analyses.

#### 2.9.7 Sub-group analyses

No sub-group analyses are planned for this study.

#### 2.9.8 Analysis of secondary outcomes

A similar modelling approach to that described for the primary outcome will be used to estimate between-arm differences for continuous secondary outcomes. For secondary outcomes that are counts, between-arm differences will be estimated using mixed effects negative binomial regression models. The specification of random and fixed effects will be consistent with the modelling approach used for the primary outcome. Both unadjusted and adjusted effect estimates, 95 % confidence intervals and p-values will be presented. If the negative binomial model fails to converge or is not an appropriate fit based on standard diagnostic measures (e.g. overdispersion checks, residual analysis etc.), alternative models such as mixed effects Poisson regression or zero-inflated models will be considered, depending on the observed distribution. For ordinal secondary outcomes, trial arm differences will be assessed using Kruskal-Wallis statistical tests. All results for secondary outcomes will be presented as shown in Appendix A Supplementary File 1 (Table 2, Table 3).

HypoPAST is hypothesised to positively impact various psychological, behavioural and clinical outcomes. For this reason, several secondary outcomes were specified. The analysis of these secondary outcomes is considered exploratory and therefore no formal adjustments for multiplicity will be conducted. However, interpretation of results relating to the secondary outcomes will take into account the strength of evidence and consistency of effects.

#### 2.9.9 Analysis of safety outcomes

The number and proportion of participants experiencing at least one SAE, and median number of SAEs will be summarised by trial arm (Table 4). The data will also be presented by SAE type (i.e. severe hypoglycaemic event or mental health-related event requiring medical assistance for recovery, defined as a call for emergency services, attendance at an emergency department, and/or admission to hospital).

**Table 4:** Safety outcomes.

	Total (N = )	Intervention (N = )	Control (N = )
**At least one SAE, *n(%)***			
**Median number of SAEs, *median [IQR]***			
**Type of SAE: Severe hypoglycaemic event**			
Emergency services were called			
*n(%)*			
*Median [IQR]*			
Taken to emergency department			
*n(%)*			
*Median [IQR]*			
Admitted to hospital			
*n(%)*			
*Median [IQR]*			
**Type of SAE: Mental health event**			
Emergency services (e.g. ambulance) or Crisis Assessment and Treatment Team were called			
*n(%)*			
*Median [IQR]*			
Taken to emergency department			
*n(%)*			
*Median [IQR]*			
Admitted to hospital			
*n(%)*			
*Median [IQR]*			

Abbreviations: SAE = serious adverse event, IQR = interquartile range.

## 3 Discussion

This statistical analysis plan outlines the methods that will be used to assess the effect of HypoPAST on fear of hypoglycaemia and other psychological, behavioural and clinical outcomes among adults with type 1 diabetes. Given the lack of evidence or consensus for a minimum clinically important difference in the primary outcome (HFS-II Worry scale score), a range of sample sizes were considered for this trial. The final estimated sample size was chosen by considering standardised effects sizes, achievable recruitment numbers and anticipated attrition rates, based on our research team's prior experience conducting trials among adults with type 1 diabetes.

More than 5000 simulations of the randomisation process were conducted to determine the optimal randomisation block sizes that would minimise imbalance in trial arm allocation within each of the 4 strata. Of the various combinations of block sizes [2], [4], [6], [8] trialled, randomly permuted block sizes of 4 and 6 minimised the mean imbalance and estimated proportion of simulations with imbalance.

Lastly, to test the robustness of our trial results, we have prespecified two sensitivity analyses. One will account for missing data that may be missing at random, and another will test the assumption that missing data are missing at random. The latter will provide insight into the possible range of the intervention effect should the unobserved data be not missing at random.

This statistical analysis plan was written prior to locking the HypoPAST trial database. All analysis methods were prespecified and are not driven by the study data. The trial outcomes paper will be based on the statistical framework outlined in this plan and available upon completion of data collection and analysis, which is anticipated in the second quarter of 2025.

## CRediT authorship contribution statement

**Sharmala Thuraisingam:** Writing – original draft, Methodology, Conceptualization. **Jennifer A. Halliday:** Writing – review & editing, Methodology, Conceptualization. **Uffe Søholm:** Writing – review & editing, Methodology, Conceptualization. **Elizabeth Holmes-Truscott:** Writing – review & editing, Conceptualization. **Christel Hendrieckx:** Writing – review & editing, Conceptualization. **Timothy C. Skinner:** Writing – review & editing, Conceptualization. **Vincent L. Versace:** Writing – review & editing, Methodology, Conceptualization. **Jane Speight:** Writing – review & editing, Methodology, Conceptualization.

## Data availability

No data were used for this statistical analysis plan.

## Funding source

The HypoPAST randomised controlled trial is supported by the Medical Research Future Fund (MRFF) Targeted Translation Research Accelerator (TTRA). In-kind support is provided by 10.13039/501100022904Australian Diabetes Educators Association, 10.13039/501100001065Australian Diabetes Society, Diabetes Australia, Diabetes Victoria, and uMotif, and academic partners: 10.13039/501100001778Deakin University, 10.13039/501100001215La Trobe University, 10.13039/501100001779Monash University, and 10.13039/501100000774Newcastle University. EH-T, CH and JSp are supported by the core funding to the Australian Centre for Behavioural Research in Diabetes (ACBRD) provided by the collaboration between Diabetes Victoria and 10.13039/501100001778Deakin University. VLV is supported by the Rural Health Multidisciplinary Training (RHMT) training programme (funded by the 10.13039/100015539Australian Government Department of Health, Disability and Aged Care). We thank 10.13039/501100000774Newcastle University for permission to use the ‘my hypo compass’ psycho-educational program to inform the generation of the novel HypoPAST programme, and acknowledge the peer-reviewed funding from 10.13039/501100000361Diabetes UK, provided to 10.13039/501100000774Newcastle University, which enabled creation of ‘my hypo compass’ as part of the HypoCOMPaSS study.

## Declaration of competing interest

The authors declare that they have no known competing financial interests or personal relationships that could have appeared to influence the work reported in this paper.
