# Supplementary material for: Statistical analysis plan for the 24-week randomised trial of hypoglycaemia prevention, awareness of symptoms, and treatment: HypoPAST
Source: Contemp Clin Trials Commun. 2025 Jul 2;46:101513. doi: 10.1016/j.conctc.2025.101513 (PMC12271082; doi:10.1016/j.conctc.2025.101513)
Supplement: Multimedia component 1 [file mmc1.docx]

**Supplementary File 1.**

**Table 1. Scoring for primary and secondary outcome measures.**

| **Measure** | **Items** | **Responses** | **Scoring** | **Interpretation of score or definition of measure** |
| --- | --- | --- | --- | --- |
|  |  |  |  |  |
| ***Glucose levels, targets, and monitoring*** |  |  |  |  |
| Most recent HbA1c (% or mmol/mol) | 1 | Numeric | N/A | HbA1c, also known as glycated haemoglobin, is a measurement of average blood glucose levels over the past 2-3 months. It is measured and discussed routinely (usually every 3-6 months) as part of diabetes care. Higher HbA1c levels indicate higher blood glucose levels over the past 2-3 months, which is an indicator for risk of long-term complications. In this study, HbA1c is self-reported. Participants will be asked to enter their most recent HbA1c in either % or mmol/mol. They will be instructed to leave this question blank if they are unsure. Participants will also be asked when their last HbA1c was measured.  Only HbA1c measured in the past 3 months will be used for mid- and end-trial analyses. |
| Target glucose lower and upper limits (mmol/L) | 2 | Numeric | N/A | Participants using finger pricks for checking blood glucose levels will be asked to provide a lower and upper value for their target glucose range. Participants using CGM/isCGM will be asked to provide their target lower and upper glucose values as set in their glucose monitoring device. |
| Time below and above target range (%; CGM/isCGM users only) | 2 | Numeric | N/A | Only participants using CGM/isCGM will be asked to provide values from their glucose monitoring device for percentage of time spent below and above their target glucose range. The exact methods used to estimate these percentages will be dependent on the device used but typically involves calculating the proportion of glucose readings that fall outside the predefined target range over a specified duration. |
| Comfortable blood glucose lower and upper limits (mmol/L) | 2 | Numeric | N/A | Participants will be asked to report the lowest and highest blood glucose levels they feel comfortable with. |
| **Primary outcome measure (assessed at 24 weeks)** | | | | |
| Hypoglycaemia Fear Survey II (HFS-II) Worry subscale | 18 | Each item scored on five-point scale  (0-4). | Total score derived by summing individual items. Range=0-72. | Higher scores indicate greater worry. |
| **Secondary outcome measures** | | | | |
| ***Awareness of hypoglycaemia symptoms*** | | | | |
| Gold score | 1 | Seven-point scale (1-7). | Range=1-7 | Score of 4 or higher indicates impaired awareness of hypoglycaemia. |
| Technological awareness: Does your glucose monitor let you know when your hypos are commencing? | 1 | Seven-point scale (1-7). | Range 1-7. | Lower scores indicate glucose monitor informing of hypoglycaemia more frequently. |
| Hypoglycaemia Awareness Questionnaire (HypoA-Q) Impaired Awareness subscale | 5 | Each item scored on five-point scale  (0-4). | Range 0-20 | Higher scores indicate lower blood glucose thresholds required for experience of symptoms. |
| ***Behaviours associated with fear of hypoglycaemia*** | | | | |
| Hypoglycaemia Fear Survey (HFS-II Short Form) – Avoidance subscale | 3 | Each item scored on five-point scale (0-4). | Score derived by summing individual items. Range=0-12. | Higher scores indicate higher frequency of hypoglycaemia avoidance behaviours. |
| Hypoglycaemia Fear Survey (HFS-II Short Form) – Maintain high subscale | 2 | Each item scored on five-point scale (0-4). | Score derived by summing individual items. Range=0-8. | Higher scores indicate higher frequency of behaviours relating to maintaining high blood glucose. |
| ***Confidence in managing hypoglycaemia*** | | | | |
| Hypoglycaemia Confidence Scale | 9 | Each item scored on four-point scale (1-4). | Item scores averaged. Range 1-4. | Scores of 3 or greater indicate at least moderate confidence. |
| ***Hypoglycaemia-specific quality of life*** | | | | |
| Hypoglycaemia-specific quality of life (HIP-12) | 12 | Each item scored on seven-point scale (1-7). | Composite score derived by averaging answered items. A composite score cannot be calculated if there are more than 7 missing items.  Range=1-7. | Composite scores:  >4 = negative impact of hypoglycaemia on quality of life  4 = no impact  <4 = positive impact of hypoglycaemia on quality of life. |
| ***Attitudes to awareness***  Attitudes to Awareness of Hypoglycaemia scale (A2A) | | | |  |
| How concerned are you about having “impaired awareness”? (If scored ≥4 on Gold score) | 1 | Categorical (5 levels ‘not at all’-‘extremely’)  Categorical (5 levels ‘not at all’-‘extremely’) | - | - |
| How motivated do you feel to get your hypo awareness (warning signs) back? (If scored ≥4 on Gold score) | 1 |  | - | - |
| ‘Asymptomatic hypoglycaemia normalised’ factor | 4 | Each item scored on four-point scale (0-3). | Sum of individual items 6,7,10 and 15. | Higher scores indicate greater normalisation of asymptomatic hypoglycaemia |
| 'Hyperglycaemia avoidance prioritised’ factor | 4 | Each item scored on four-point scale (0-3). | Sum of individual items 8,12,16 and 19. | Higher scores indicate greater prioritisation of avoidance of hyperglycaemia |
| ‘Hypoglycaemia concern minimised’ factor | 4 | Each item scored on four-point scale (0-3). | Sum of individual items 11,14,17 and 18. | Higher scores indicate greater minimisation of concerns about hypoglycaemia |
| I don’t need to worry about hypos because I don’t get them very often | 1 | Categorical (4 levels ‘not true at all’-‘very true’)  Categorical (5 levels ‘not at all’-‘extremely’) | - | - |
| I’m not too bothered about warning signs because severe hypos are rare for me | 1 |  |  |  |
| ***Perceptions and experiences of hypoglycaemia*** |  |  |  |  |
| Hypoglycaemia Cues Questionnaire (Hypo C-Q) |  |  |  |  |
| Low concern about hypoglycaemia scale | 7 | Each item scored on five-point likert scale (1-5). | Mean of items 1B,2B,3B,7B,8B,9B,14B | Higher scores indicate fewer concerns related to hypoglycaemia |
| Hypoglycaemia burnout scale | 6 |  | Mean of items 5B,6B,10B,11B,12B,13B | Higher scores indicate higher hypoglycaemia burnout |
| Missing opportunities to treat hypoglycaemia scale | 5 |  | Mean of items 7C,8C,10C,11C,12C | Higher scores indicate experience of more missed opportunities to treat hypoglycaemia |
| Delaying treatment of hypoglycaemia scale | 9 |  | Mean of items 2D*,3D,4D,5D,6D,8D,9D | Higher scores indicate more frequently delaying treatment of hypoglycaemia |
| Individual questions (12 items) | 12 |  | - | - |
| ***Hypoglycaemia-specific post-traumatic stress*** | | | | |
| Primary Care Post Traumatic Stress Disorder Screen for Diagnostic of Statistical Manual of Mental Disorders 5^th^ Edition adapted for hypoglycaemia (PC-PTSD-5) | 5 | Each item scored as 0=no, 1=yes. | Total score derived by summing individual items. | Higher scores indicate more traumatic stress in relation to past episodes of severe hypoglycaemia. |
| ***Diabetes distress*** |  |  |  |  |
| Problem Areas in Diabetes (PAID-11) score | 11 | Each item scored on five-point scale (0-4). | Total score derived by summing individual items. Range=0-44 | Higher scores indicate greater diabetes distress. |
| ***Anxiety and depressive symptoms*** | | | | |
| Patient Health Questionnaire (PHQ-4) anxiety subscale | 2 | Each item scored on four-point scale (0-3). | Subscale score derived by summing responses to questions 1-2. | A subscale score of 3 or greater may indicate anxiety/depressive symptoms. |
| Patient Health Questionnaire (PHQ-4) depression subscale | 2 |  | Subscale score derived by summing responses to questions 3-4. |  |

All primary and secondary outcome measures are self-reported. Items marked as * need to be reversed when including in total score.

**Table 2. Estimated trial arm differences in continuous and count secondary outcomes**

|  | **Intervention (N=)** | | | **Control (N=)** | | | **Unadjusted** | | | **Adjusted** | |
| --- | --- | --- | --- | --- | --- | --- | --- | --- | --- | --- | --- |
|  | **N** | **Mean (SD)** | **Estimated mean (95% CI)** | **N** | **Mean (SD)** | **Estimated mean (95% CI)** | **Intervention vs control between-arm differences (95% CI)** | | **P-value** | **Intervention vs control between-arm differences (95% CI)** | **P-value** |
| ***Glucose levels, targets, and monitoring*** |  |  |  |  |  |  |  | |  |  |  |
| Most recent HbA1c (%) self-reported |  |  |  |  |  |  |  | |  |  |  |
| Baseline |  |  |  |  |  |  |  | |  |  |  |
| Mid-trial |  |  |  |  |  |  |  | |  |  |  |
| End-trial |  |  |  |  |  |  |  | |  |  |  |
| Target glucose lower limit (mmol/L) |  |  |  |  |  |  |  | |  |  |  |
| Baseline |  |  |  |  |  |  |  | |  |  |  |
| Mid-trial |  |  |  |  |  |  |  | |  |  |  |
| End-trial |  |  |  |  |  |  |  | |  |  |  |
| Target glucose upper limit (mmol/L) |  |  |  |  |  |  |  | |  |  |  |
| Baseline |  |  |  |  |  |  |  | |  |  |  |
| Mid-trial |  |  |  |  |  |  |  | |  |  |  |
| End-trial |  |  |  |  |  |  |  | |  |  |  |
| Time below target range (CGM/isCGM) (%) |  |  |  |  |  |  |  | |  |  |  |
| Baseline |  |  |  |  |  |  |  | |  |  |  |
| Mid-trial |  |  |  |  |  |  |  | |  |  |  |
| End-trial |  |  |  |  |  |  |  | |  |  |  |
| Time above target range (CGM/isCGM) (%) |  |  |  |  |  |  |  | |  |  |  |
| Baseline |  |  |  |  |  |  |  | |  |  |  |
| Mid-trial |  |  |  |  |  |  |  | |  |  |  |
| End-trial |  |  |  |  |  |  |  | |  |  |  |
| Comfortable blood glucose lower limit (mmol/L) |  |  |  |  |  |  |  | |  |  |  |
| Baseline |  |  |  |  |  |  |  | |  |  |  |
| Mid-trial |  |  |  |  |  |  |  | |  |  |  |
| End-trial |  |  |  |  |  |  |  | |  |  |  |
|  |  |  |  |  |  |  |  | |  |  |  |
|  | **Intervention (N=)** | | | **Control (N=)** | | | **Unadjusted** | | | **Adjusted** | |
|  | **N** | **Mean (SD)** | **Estimated mean (95% CI)** | **N** | **Mean (SD)** | **Estimated mean (95% CI)** | **Intervention vs control between-arm differences (95% CI)** | **P-value** | | **Intervention vs control between-arm differences (95% CI)** | **P-value** |
| Comfortable blood glucose upper limit (mmol/L) |  |  |  |  |  |  |  |  | |  |  |
| Baseline |  |  |  |  |  |  |  |  | |  |  |
| Mid-trial |  |  |  |  |  |  |  |  | |  |  |
| End-trial |  |  |  |  |  |  |  |  | |  |  |
|  | **Intervention (N=)** | | |  | **Control (N=)** | | **Unadjusted** | | | **Adjusted** | |
| ***Insulin use, self-reported daily insulin units*** | **N** | **Median [IQR]** | | **N** | **Median [IQR]** | | **IRR (95% CI)** | **P-value** | | **IRR (95% CI)** | **P-value** |
| Baseline |  |  |  |  |  |  |  | |  |  |  |
| Mid-trial |  |  |  |  |  |  |  | |  |  |  |
| End-trial |  |  |  |  |  |  |  | |  |  |  |
|  | **Intervention (N=)** | | |  | **Control (N=)** | | **Unadjusted** | | | **Adjusted** | |
| ***Awareness of hypoglycaemia symptoms*** | **N** | **Mean (SD)** | **Estimated mean (95% CI)** | **N** | **Mean (SD)** | **Estimated mean (95% CI)** | **Intervention vs control between-arm differences (95% CI)** | | **P-value** | **Intervention vs control between-arm differences (95% CI)** | **P-value** |
| Gold score |  |  |  |  |  |  |  | |  |  |  |
| Baseline |  |  |  |  |  |  |  | |  |  |  |
| Mid-trial |  |  |  |  |  |  |  | |  |  |  |
| End-trial |  |  |  |  |  |  |  | |  |  |  |
| Technology awareness |  |  |  |  |  |  |  | |  |  |  |
| Baseline |  |  |  |  |  |  |  | |  |  |  |
| Mid-trial |  |  |  |  |  |  |  | |  |  |  |
| End-trial |  |  |  |  |  |  |  | |  |  |  |
| HypoA-Q Impaired Awareness subscale |  |  |  |  |  |  |  | |  |  |  |
| Baseline |  |  |  |  |  |  |  | |  |  |  |
| Mid-trial |  |  |  |  |  |  |  | |  |  |  |
| End-trial |  |  |  |  |  |  |  | |  |  |  |
|  |  | **Intervention (N=)** | |  | **Control (N=)** | | **Unadjusted** | | | **Adjusted** | |
| ***Hypoglycaemia frequency & severity*** |  | **N** | **Median [IQR]** |  | **N** | **Median [IQR]** | **IRR (95% CI)** | | **P-value** | **IRR (95% CI)** | **P-value** |
| Number of hypoglycaemic episodes (mild or severe) in past week |  |  |  |  |  |  |  | |  |  |  |
| Baseline |  |  |  |  |  |  |  | |  |  |  |
| End-trial |  |  |  |  |  |  |  | |  |  |  |
| Number of severe hypoglycaemic episodes in past 6 months |  |  |  |  |  |  |  | |  |  |  |
| Baseline |  |  |  |  |  |  |  | |  |  |  |
| End-trial |  |  |  |  |  |  |  | |  |  |  |
|  | **Intervention (N=)** | | | **Control (N=)** | | | **Unadjusted** | | | **Adjusted** | |
| ***Behaviours associated with fear of hypoglycaemia*** | **N** | **Mean (SD)** | **Estimated mean (95% CI)** | **N** | **Mean (SD)** | **Estimated mean (95% CI)** | **Intervention vs control between-arm differences (95% CI)** | | **P-value** | **Intervention vs control between-arm differences (95% CI)** | **P-value** |
| Fear of hypoglycaemia (HFS-II) |  |  |  |  |  |  |  | |  |  |  |
| Avoidance subscale |  |  |  |  |  |  |  | |  |  |  |
| Baseline |  |  |  |  |  |  |  | |  |  |  |
| Mid-trial |  |  |  |  |  |  |  | |  |  |  |
| End-trial |  |  |  |  |  |  |  | |  |  |  |
| Maintain high subscale |  |  |  |  |  |  |  | |  |  |  |
| Baseline |  |  |  |  |  |  |  | |  |  |  |
| Mid-trial |  |  |  |  |  |  |  | |  |  |  |
| End-trial |  |  |  |  |  |  |  | |  |  |  |
| ***Confidence in managing hypoglycaemia (hypoglycaemia confidence scale)*** |  |  |  |  |  |  |  | |  |  |  |
| Baseline |  |  |  |  |  |  |  | |  |  |  |
| Mid-trial |  |  |  |  |  |  |  | |  |  |  |
| End-trial |  |  |  |  |  |  |  | |  |  |  |
| ***Hypoglycaemia-specific quality of life (HIP-12)*** |  |  |  |  |  |  |  | |  |  |  |
| Baseline |  |  |  |  |  |  |  | |  |  |  |
| Mid-trial |  |  |  |  |  |  |  | |  |  |  |
| End-trial |  |  |  |  |  |  |  | |  |  |  |
| ***Attitudes to awareness of hypoglycaemia (A2A)*** |  |  |  |  |  |  |  | |  |  |  |
| ‘Asymptomatic hypoglycaemia normalised’ factor |  |  |  |  |  |  |  | |  |  |  |
| Baseline |  |  |  |  |  |  |  | |  |  |  |
| Mid-trial |  |  |  |  |  |  |  | |  |  |  |
| End-trial |  |  |  |  |  |  |  | |  |  |  |
| ‘Hyperglycaemia avoidance prioritised’ factor |  |  |  |  |  |  |  | |  |  |  |
| Baseline |  |  |  |  |  |  |  | |  |  |  |
| Mid-trial |  |  |  |  |  |  |  | |  |  |  |
| End-trial |  |  |  |  |  |  |  | |  |  |  |
| ‘Hypoglycaemia concern minimised’ factor |  |  |  |  |  |  |  | |  |  |  |
| Baseline |  |  |  |  |  |  |  | |  |  |  |
| Mid-trial |  |  |  |  |  |  |  | |  |  |  |
| End-trial |  |  |  |  |  |  |  | |  |  |  |
| ***Perceptions and experiences of hypoglycaemia (Hypo C-Q)*** |  |  |  |  |  |  |  | |  |  |  |
| Low concern about hypoglycaemia scale |  |  |  |  |  |  |  | |  |  |  |
| Baseline |  |  |  |  |  |  |  | |  |  |  |
| Mid-trial |  |  |  |  |  |  |  | |  |  |  |
| End-trial |  |  |  |  |  |  |  | |  |  |  |
| Hypoglycaemia burnout scale |  |  |  |  |  |  |  | |  |  |  |
| Baseline |  |  |  |  |  |  |  | |  |  |  |
| Mid-trial |  |  |  |  |  |  |  | |  |  |  |
| End-trial |  |  |  |  |  |  |  | |  |  |  |
| Missing opportunities to treat hypoglycaemia scale |  |  |  |  |  |  |  | |  |  |  |
| Baseline |  |  |  |  |  |  |  | |  |  |  |
| Mid-trial |  |  |  |  |  |  |  | |  |  |  |
| End-trial |  |  |  |  |  |  |  | |  |  |  |
| Delaying treatment of hypoglycaemia scale |  |  |  |  |  |  |  | |  |  |  |
| Baseline |  |  |  |  |  |  |  | |  |  |  |
| Mid-trial |  |  |  |  |  |  |  | |  |  |  |
| End-trial |  |  |  |  |  |  |  | |  |  |  |
| ***Hypoglycaemia-related post-traumatic stress (PC-PTSD-5a)*** |  |  |  |  |  |  |  | |  |  |  |
| Baseline |  |  |  |  |  |  |  | |  |  |  |
| Mid-trial |  |  |  |  |  |  |  | |  |  |  |
| End-trial |  |  |  |  |  |  |  | |  |  |  |
| ***Diabetes distress (PAID scale)*** |  |  |  |  |  |  |  | |  |  |  |
| Baseline |  |  |  |  |  |  |  | |  |  |  |
| Mid-trial |  |  |  |  |  |  |  | |  |  |  |
| End-trial |  |  |  |  |  |  |  | |  |  |  |
| ***Anxiety and depressive symptoms (PHQ-4)*** |  |  |  |  |  |  |  | |  |  |  |
| Anxiety symptoms |  |  |  |  |  |  |  | |  |  |  |
| Baseline |  |  |  |  |  |  |  | |  |  |  |
| Mid-trial |  |  |  |  |  |  |  | |  |  |  |
| End-trial |  |  |  |  |  |  |  | |  |  |  |
| Depressive symptoms |  |  |  |  |  |  |  | |  |  |  |
| Baseline |  |  |  |  |  |  |  | |  |  |  |
| Mid-trial |  |  |  |  |  |  |  | |  |  |  |
| End-trial |  |  |  |  |  |  |  | |  |  |  |

All primary and secondary outcome measures are self-reported.

Unadjusted=Adjusted by stratification factors gender and blood glucose monitoring method only. Adjusted=Adjusted by stratification factors (gender, blood glucose monitoring method), age, diabetes duration, HbA1c, severe hypoglycaemia in the last 6 months, Gold score and insulin administration modality where relevant. Abbreviations: SD=standard deviation, CI=confidence interval, HbA1c=Glycated haemoglobin, CGM=continuous glucose monitoring, isCGM=intermittently scanned continuous glucose monitor (Flash), IQR=interquartile range, IRR=incident rate ratio

### **Table 3. Statistical tests for ordinal secondary outcomes**

|  |  |  |
| --- | --- | --- |
|  | **η^2^ (Kruskal-Wallis)** | **P-value** |
| **Glucose checks** |  |  |
| Daily number of glucose checks (isCGM/CGM) |  |  |
| Mid-trial |  |  |
| End-trial |  |  |
| Daily number of glucose checks (finger prick) |  |  |
| Mid-trial |  |  |
| End-trial |  |  |
| **Insulin use** |  |  |
| Daily number of insulin doses/boluses |  |  |
| Mid-trial |  |  |
| End-trial |  |  |
| **Hypoglycaemia symptom burden & response** |  |  |
| My hypo symptoms bother me^+^ |  |  |
| Mid-trial |  |  |
| End-trial |  |  |
| My hypo symptoms help me to identify and treat my hypo early^+^ |  |  |
| Mid-trial |  |  |
| End-trial |  |  |
| **Hypoglycaemia frequency & severity** |  |  |
| Frequency of hypoglycaemia in past 6 months when awake (End-trial) |  |  |
| Able to treat yourself |  |  |
| Unable to treat yourself |  |  |
| Needed someone else to give you sugar by mouth |  |  |
| Needed someone else to give you a glucagon injection |  |  |
| Frequency of hypoglycaemia in past 6 months when asleep (End-trial) |  |  |
| Unable to treat yourself when you woke |  |  |
| Someone else gave you sugar by mouth |  |  |
| Someone else gave you a glucagon injection |  |  |
| Led to a major problem (e.g fit, fall etc.) |  |  |
| Stayed asleep, later realised had a hypo |  |  |
| **Attitudes to awareness (A2A)** |  |  |
| How concerned are you about having “impaired awareness”?** |  |  |
| Midtrial |  |  |
| End trial |  |  |
| How motivated do you feel to get your hypo awareness (warning signs) back?** |  |  |
| Mid-trial |  |  |
| End-trial |  |  |
| I don’t need to worry about hypos because I don’t get them very often |  |  |
| Mid-trial |  |  |
| End-trial |  |  |
| I’m not too bothered about warning signs because severe hypos are rare for me |  |  |
| Mid-trial |  |  |
| End-trial |  |  |

All secondary outcome measures are self-reported.

+Only applies to participants who reported having symptoms when their blood glucose is low on the Hypo A-Q Impaired Awareness subscale

**If scored 4 or higher on the Gold score

Abbreviations: CGM=continuous glucose monitoring, A2A=Attitudes to awareness of hypoglycaemia
